# First documented case of a fatal autochthonous Usutu virus infection in an immunocompromised patient in Hungary: a clinical-virological report and implications from the literature

**DOI:** 10.1186/s12985-025-02890-9

**Published:** 2025-07-29

**Authors:** Bálint Gergely Szabó, Anna Nagy, Orsolya Nagy, Anita Koroknai, Nikolett Csonka, Dorina Korózs, Krisztina Jeszenszky, Apor Hardi, Nóra Deézsi-Magyar, János Sztikler, Zoltán Bódi, Dániel Cadar, Gábor Endre Tóth, Liliána Veres, Erzsébet Barcsay, Mária Takács, János Sinkó

**Affiliations:** 1 Departmental Group of Infectious Diseases, Department of Internal Medicine and Haematology, Semmelweis University, Szentkirályi utca 46, Budapest, 1088 Hungary; 2 Doctoral College, Semmelweis University, Üllői út 26, Budapest, 1085 Hungary; 3 South Pest Central Hospital, National Institute of Hematology and Infectious Diseases, Albert Flórián út 5-7, Budapest, H-1097 Hungary; 4 National Reference Laboratory for Viral Zoonoses, National Center for Public Health and Pharmacy, Albert Flórián út 2-6, Budapest, 1097 Hungary; 5 Institute of Medical Microbiology, Semmelweis University, Nagyvárad tér 4, Budapest, 1089 Hungary; 6 National Biosafety Laboratory, National Center for Public Health and Pharmacy, Albert Flórián út 2-6, Budapest, 1097 Hungary; 7 Department of Bacteriological, Mycological and Parasitological Reference Laboratory, National Center for Public Health and Pharmacy, Albert Flórián út 2-6, Budapest, 1097 Hungary; 8 Bernhard Nocht Institute for Tropical Medicine, Bernhard-Nocht-Straße 74), Hamburg, 20359 Germany; 9 Faculty of Medicine, Semmelweis University, Budapest, 1085 Hungary; 10 Department of Virology, National Center for Public Health and Pharmacy, Albert Flórián út 2-6, Budapest, 1097 Hungary

**Keywords:** Usutu virus, USUV, Orthoflavivirus, Zoonotic virus, Immunocompromised host

## Abstract

**Background:**

Usutu virus (USUV) is a mosquito-borne neurotropic orthoflavivirus, endemic to Europe. Although incidental human infections have been recognized, comprehensive descriptions remain scarce. Herein, we report the clinical-virological analysis of the first documented autochthonous case of fatal USUV infection in a severely immunocompromised adult from Hungary.

**Clinical presentation:**

A 61-year-old female with relapsed acute myelomonocytic leukemia developed progressive neurological symptoms, accompanied by high-grade fever, during post-chemotherapy aplasia. Initial cranial MRI revealed symmetric thalamic and brainstem abnormalities, while cerebrospinal fluid analysis showed mildly elevated protein levels. Despite empirical antimicrobial therapy, her status deteriorated with new-onset dysarthria and somnolence by day + 29 post-chemotherapy, requiring admission to the intensive care unit. Subsequent EEG demonstrated diffuse background slowing, and follow-up MRI confirmed further progression of the lesions. Despite supportive care and extensive microbiological testing, the patient died on day + 37 post-chemotherapy.

**Virological investigation:**

USUV RNA was detected in CSF, blood, urine, and *post-mortem* tissues by RT-qPCR, using validated *in-house* protocols. Virus isolation was successfully achieved *via* intracranial inoculation of newborn mice and subsequent culture in Vero E6 cell cultures. Whole-genome sequencing and phylogenetic analysis confirmed infection with the USUV Europe 2 lineage, closely related to other Hungarian and Italian strains. No other pathogens from the central nervous system were identified.

**Conclusions:**

We highlight the challenges of USUV infection in immunocompromised patients. The phylogenetic link between European strains shows the regional emergence of high-risk viral lineages. Surveillance, donor screening, and research into antiviral therapies are needed to mitigate the impact of this emerging arbovirus.

**Supplementary Information:**

The online version contains supplementary material available at 10.1186/s12985-025-02890-9.

## Introduction

Usutu virus (USUV) is a neurotropic mosquito-borne orthoflavivirus within the family *Flaviviridae*, genus *Orthoflavivirus* [1]. USUV belongs to the Japanese encephalitis virus (JEV) serogroup within the orthoflavivirus genus, sharing cross-reactive neutralizing antibodies with other members of the JEV-serocomplex, such as West Nile virus (WNV) [1]. The zoonotic potential of the virus was first described in Africa in the 1980s, with the first human USUV infection reported in 1981 in the Central African Republic [2]. The patient presented with fever and rash [2]. Birds are responsible for the ongoing maintenance of the enzootic cycle of the virus as amplifying hosts, while ornithophilic mosquitoes act as vectors. USUV was first isolated from *Culex naevei* in 1959 near the Maputo River (also called the Usutu River) in South Africa [3]. Since then, the virus has been detected in various native and invasive mosquito species of Hungary, although its main vector is the *Culex pipiens* [4]. USUV can be transmitted to humans or other mammals (such as horses) via mosquito bites; however, humans are unable to produce an appropriate level of viremia for vector-borne human-to-human transmission [5]. Therefore, humans are considered incidental or “dead-end” hosts, but virus transmission between humans by blood transfusion or organ transplantation cannot be excluded. Although the clinical consequences of receiving USUV-positive blood or blood products are still unknown, the presence of the virus in blood donors has already been demonstrated [6–9]. Different genetic lineages of USUV have emerged in recent years, due to the multiple introduction events to Europe from Africa, facilitated by the migration of its avian hosts. According to phylogenetic analysis, USUV strains were clustered into eight genetic lineages: three African (Africa 1–3) and five European (Europe 1–5) [10].

The first two autochthonous human infections in Europe were detected in Italy in 2009 [11]. Both patients were immunocompromised and suffered from meningoencephalitis [11, 12]. Since then, over a hundred cases of acute human infections have been reported in Europe [13]. The majority of human infections are asymptomatic or associated with mild symptoms such as moderate fever, fatigue, and maculopapular exanthema [4, 5]. The neuroinvasive nature of the virus has been confirmed in murine models [14, 15]. According to a comprehensive review by *Cadar and Simonin*, at least 31 human USUV cases characterized by neurological disorders had been reported until 2022 [16]. The co-circulation of USUV and WNV in Hungary is well-known; however, USUV-related clinical data remains limited [17, 18]. As part of the avian monitoring program, USUV was first detected in a dead *Turdus merula* in Budapest in 2005 [19]. The first human USUV infection associated with aseptic meningitis was reported in 2018 [17]. Although parallel screening for USUV and WNV is part of the routine differential diagnostic algorithm during the mosquito season, no further human USUV infections were confirmed between 2019 and 2023.

During the 2024 transmission season, the number of reported WNV cases in Hungary remarkably exceeded the usual annual case numbers of recent years [20]. Along with the emergence of WNV, increased USUV activity was also observed. In total, three human USUV infections have been confirmed by virological methods, including the present case of an immunocompromised patient with a fatal outcome. As the current knowledge about USUV infection among immunocompromised patients is substantially limited, our aim was to present a detailed clinical and laboratory description of the fatal USUV infection case diagnosed in 2024 and provide a brief overview of the literature on this emerging threat.

## Case investigation

### Clinical presentation

A 61-year-old Caucasian female with a medical history of Vater papilla adenocarcinoma in complete remission was admitted for FLAG-M salvage chemotherapy (fludarabine 50 mg/day on days + 1 to + 5, cytarabine 1700 mg/day on days + 1 to + 5, mitoxantrone 10 mg/day on day + 1, and 15 mg/day on days + 2 to + 3) due to relapsed acute myelomonocytic leukemia (AMMoL), which had previously transformed from adverse-risk chronic myelomonocytic leukemia (Table 1). On day + 7 following chemotherapy initiation, the patient developed chemotherapy-induced aplasia. By day + 22, she presented with new-onset febrile neutropenia, requiring the initiation of empirical ceftazidime.

**Table 1:** Clinical characteristics

Parameter	**Time since chemotherapy initiation**						
	Day + 25	Day + 27	Day + 29	Day + 31	Day + 33	Day + 35	Day + 37
**Site of hospitalization**	Hematology department	Hematology department	Hematology department, and ICU	ICU	ICU	ICU	ICU
Fever*	Yes	Yes	Yes	Yes	Yes	Yes	Yes
**Glasgow coma scale** (eye/verbal/motor)	4/5/6	4/4/6	3/4/5	3/3/2^**^	1/T/1	1/T/1	1/T/1
**Neurological symptoms**:					NA	NA	NA
- Dysphagia				Yes			
- Dysarthria			Yes	Yes			
- Diplopia	Yes	Yes	Yes	Yes			
- Limb ataxia		Yes	Yes	Yes			
**Cranial MRI performed**	Yes		Yes		Yes		
**CSF analysis**:							
- White blood cell count (cell/ul)	1		1		2		
- Protein level (g/L)	0.5		0.9		1.3		
- Glucose level (mmol/L)	2.5		3.8		3.6		
**Laboratory analysis**:							
- Blood neutrophil level (×10^9^/L)	0	0	0	0	0	0	0
- Blood hemoglobin level (g/L)	93	83	80	77	92	93	89
- Blood thrombocyte level (×10^9^/L)	28	14	11	16	11	5	11
- Serum creatinine (µmol/L)	55	53	56	78	83	137	186
- Serum C-reactive protein (mg/L)	11	18	26	58	75	270	259
- Serum procalcitonin (ng/mL)	0.18	0.21	0.16	0.18	0.38	22.1	39.5
- Plasma interleukin-6 (pg/mL)	n.a.	14.5	38.2	94.8	102.1	3652.3	54573.4
**Respiratory support**	Low-flow nasal cannula	Venturi mask (FiO_2_ 50%)	Venturi mask (FiO_2_ 50%)	Invasive mechanical ventilation	Invasive mechanical ventilation	Invasive mechanical ventilation	Invasive mechanical ventilation
**Antibiotic therapy**:							
- Ceftazidime	2 g TID	2 g TID					
- Meropenem			2 g TID	2 g TID	2 g TID	2 g TID	
**-** Levofloxacin			500 mg BID	500 mg BID	500 mg BID		
**Antifungal therapy**:							
- Liposomal amphotericin-B	300 mg QD	300 mg QD	300 mg QD	300 mg QD	300 mg QD	300 mg QD	300 mg QD
**Antiviral therapy**							
- Remdesivir			200 mg QD	100 mg QD	100 mg QD	100 mg QD	
- IVIG			30 g QD	30 g QD			

^*^Body temperature ≥ 38.0 °C (100.4 °F), measured with a tympanal noncontact thermometer at 7 AM each day

^**^Before endotracheal intubation

BID: twice per day (*bis in die*), FiO_2_: fraction of inspired oxygen, ICU: intensive care unit, IVIG: intravenous immunoglobulin, NA: not applicable, MRI: magnetic resonance imaging, QD: once per day (*quaque die*), T: intubated, TID: three times per day (*ter in die*)

**Fig. 1:**
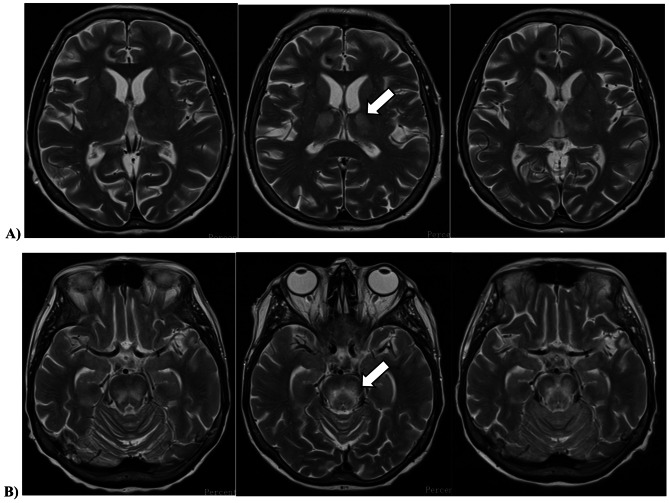
Cranial magnetic resonance imaging of the patient, showing focal progression with time. T2-weighted turbo spin echo sequences, acquired in the transverse planes of **A**) the thalamus and **B**) the midbrain

On day + 25, the patient developed new-onset diplopia, prompting an urgent cranial magnetic resonance imaging (MRI), which revealed symmetric abnormalities in the thalamus and brainstem, suggestive of metabolic encephalopathy. Multiple small foci in the brain parenchyma were consistent with microhemorrhages, though the morphology also raised suspicion of infectious foci. Differential diagnoses included Wernicke encephalopathy and osmotic demyelination syndrome (Fig. 1). A cerebrospinal fluid (CSF) analysis was unremarkable, but an elevated *Aspergillus* sp. DNA load of 471 copies/mL by polymerase-chain reaction (PCR) and a concurrently positive serum galactomannan antigen index of 3.0 by chemiluminescence immunoassay prompted for empirical antifungal therapy with liposomal amphotericin-B. Routinely used microbiological diagnostic kits for different pathogen targets are listed in the Supplementary File 1. All tests yielded negative results, with the exception of *Aspergillus* sp. A chest and paranasal CT scan was also done with unremarkable result.

On day + 29, the patient developed new-onset dysarthria and somnolence, with clear neurological deterioration. A repeat cranial MRI demonstrated significant progression of extent and intensity in the previously observed symmetric signal abnormalities in the thalamus, cerebral peduncles, brainstem tegmentum, and pons. Additionally, smaller signal abnormalities with mild progression were observed in the medulla oblongata and the craniospinal junction. A lumbar puncture was repeated, with CSF samples sent for routine bacterial/fungal culture and Gram smear, multiplex PCR for bacterial, viral and fungal pathogens, as well as CSF/blood samples *Aspergillus sp.*, *Cryptococcus sp.* and *Toxoplasma gondii* (Supplementary File 1.). In addition, a dissemination of the underlying AMMoL was ruled out by CSF flow cytometry. Additional CSF, blood, and urine samples were sent to the National Center for Public Health and Pharmacy for testing of WNV, USUV, tick-borne encephalitis (TBEV), and JC virus (JCV) (Table 2.).

**Table 2:** Results of the molecular biological assays

Sample	Sampling time after symptom onset	USUV RT-qPCR (Ct)	USUV nested RT-PCR	WNV RT-qPCR	TBEV RT-qPCR	BKV/JCV qPCR	GenBank Accession number
Serum	+ 4 days	30.57	**Positive**	Negative	Negative	Negative	NA
Whole blood	+ 4 days	33.57	**Positive**	Negative	Negative	Negative	**PV523801**
CSF	+ 4 days	31.91	**Positive**	Negative	Negative	Negative	NA
Urine	+ 4 days	33.88	**Positive**	Negative	Negative	**BKV+** **Ct 21.28**	NA
Serum	+ 11 days	32.87	NA	Negative	NA	NA	NA
Whole blood	+ 11 days	32.68	NA	Negative	NA	NA	**PV523802**
Urine	+ 11 days	33.95	NA	Negative	NA	NA	NA
Brain	+ 13 days (post-mortem)	26.94	**Positive**	Negative	NA	NA	NA
Bone marrow	+ 13 days (post-mortem)	34.02	NA	Negative	NA	NA	NA

CSF: cerebrospinal fluid, Ct: cycle threshold, BKV/JCV: BK virus/JC virus, NA: not applied, RT-qPCR: reverse transcription real-time PCR, TBEV: tick-borne encephalitis virus, USUV: Usutu virus, WNV: West Nile virus

The patient became nearly unresponsive with tachypnea and episodes of desaturation the next morning, prompting an intensive care unit (ICU) admission. A chest X-ray revealed diffuse patchy opacity in the lungs, suggestive of an infectious infiltrate. During the day, persistent desaturation and secretion retention necessitated endotracheal intubation and mechanical ventilation, while intravenous antibiotics were escalated to meropenem and levofloxacin. An extensive microbiological testing for healthcare-acquired pneumonia from bronchoalveolar lavage was done by routine bacterial/fungal culturing and Gram smear, multiplex PCR for bacterial and fungal pathogens as well as *Pneumocystis jirovecii*, *Aspergillus sp.* and CMV, with a repeated set of blood cultures (Supplementary File 1).

Given the suspicion of WNV infection due to extreme seasonal activity in the region, *off-label* empirical antiviral therapy with remdesivir and intravenous immunoglobulin (IVIG) was initiated. On the second day of ICU admittance, the National Center for Public Health and Pharmacy reported that the patient’s CSF, urine, and blood samples tested positive for Usutu virus (Table 2.). A follow-up cranial MRI revealed slight progression of previously described symmetric signal abnormalities in the thalamus and pons, while EEG showed moderate background detected in the theta range, with desynchronization in response to external stimuli, without any focal abnormalities or seizure activity. A repeat neurological consultation recommended continued supportive care, and intracranial pressure-reducing therapy was deemed unnecessary. Microbiological tests led to the discontinuation of remdesivir, and processes were initiated for the acquisition of favipiravir. Despite all efforts, the patient died on day + 37. Autopsy revealed histopathological signs of diffuse microglial activation with incipient gliosis in the brain, and diffuse alveolar damage in the lungs. Apart from USUV, no other infectious agents affecting the central nervous system (CNS) could be documented by extensive microbiological or pathological diagnostics.

### Virological investigation

Laboratory diagnosis of human arbovirus infections in Hungary is centralized and carried out exclusively at the National Reference Laboratory for Viral Zoonoses at the National Center for Public Health and Pharmacy, according to the decree of the Ministry of Health 18/1998 (VI. 3.) [21]. Serum, anticoagulant-treated whole blood, CSF, and urine samples were investigated by molecular diagnostic methods. Manual nucleic acid extraction was carried out by the QIAamp Viral RNA Mini Kit (Qiagen, Hilden, Germany), according to the manufacturer’s recommendations. Tick-borne encephalitis virus (TBEV), WNV, and USUV RNA were detected by reverse-transcription real-time PCR (RT-qPCR) method. For the WNV RNA detection, RealStar^®^ WNV CE-IVD RT-PCR Kit 2.0 (Altona Diagnostics, Hamburg-Altona, Germany) was used. TBEV and USUV RT-qPCR were performed according to *in-house* developed protocols based on primer and TaqMan probe sequences published elsewhere [22, 23]. The adapted *in-house* RT-qPCR protocols were validated prior to use with clinical specimens. The intra-assay precision was 0.78% coefficient of variation (CV) for the TBEV RT-qPCR assay and 0.69% CV for the USUV RT-qPCR assay. The limit of detection (LoD) for the TBEV RT-qPCR assay was established at a Ct value below 40.00. The USUV RT-qPCR assay demonstrated a LoD at a Ct value of 35.00, corresponding to a limit of quantification of 4 copies/µL of USUV RNA. Amplification efficiency ranged from 106 to 111%, according to the original study by *Nikolay et al.* [23]. Further differential diagnosis included testing for polyomavirus DNA using the GeneProof BK/JC Virus PCR Kit (GeneProof, Brno, Czech Republic). The results of the molecular biological investigation are summarized in Table 2. Despite the patient’s immunocompromised status, serological tests were also performed to detect TBEV, WNV, and USUV antibody response in the serum and CSF samples. For the detection of TBEV and WNV-specific IgG, IgM, and IgA antibodies, the Flavivirus Mosaic 1 (EUROIMMUN Medizinische Labordiagnostika, Lübeck, Germany) indirect immunofluorescence assay (IFA) was used. USUV-specific IgG/IgM/IgA antibody response was examined using an *in-house* developed IFA method. *In-house* USUV immunofluorescence tests were performed as described earlier [24]. Both serum and CSF samples were negative for TBEV, WNV, and USUV-specific IgG, IgM, and IgA antibodies.

### Virus isolation

In vivo virus isolation from the USUV-positive post-mortem brain specimen was performed by intracranial inoculation of newborn mice. On day 7 post-infection, mice were euthanized, and after dissection and preprocessing, the mouse brain was homogenized using MagNA Lyser Instrument (Roche Diagnostics, Basel, Switzerland). The USUV RT-qPCR result confirmed virus replication in the mouse brain, as a lower Ct value (Ct 14.29) was measured compared to the clinical specimen (Ct 26.94; Table 2.). In addition, the supernatant of the mouse brain homogenate was inoculated onto Vero E6 (ATCC; CRL-1586) cell culture. Cells were cultured for up to 7 days, and the cytopathic effect was monitored daily. USUV RT-qPCR was carried out from the cell culture supernatant (*passage 2* isolate) and confirmed the USUV replication (RT-qPCR Ct value of 14.87).

### Sequencing and phylogenetic analysis

USUV RT-qPCR results were confirmed by amplicon-based whole-genome next-generation sequencing (NGS) method. NGS was performed on samples from two patients with confirmed USUV infection in 2024, along with USUV-positive mosquito pools collected during the same period. Clinical specimens from the two patients were sent for NGS to the Bernhard Nocht Institute for Tropical Medicine, Hamburg, Germany. The NGS of mosquito pools was conducted at the National Reference Laboratory for Viral Zoonoses (Hungary). To run an amplicon-based sequencing of subsequent mosquito pools, a multiplex primer panel was designed using the Primal Scheme online tool (https://primalscheme.com). Primer sequences are listed in the Supplementary File 2. NGS was also performed on the USUV passage 2 isolate, using sequence-independent amplification of the viral RNA in this case [25].

The DNA library preparation was performed using the Nextera XT DNA Library Preparation Kit (Illumina, San Diego, CA; USA), following the manufacturer’s instructions. Sequencing was carried out on the Illumina MiSeq System (Illumina, San Diego, CA; USA) with MiSeq Reagent Micro kit, v2, 300 cycles (MS-103-1002) (Illumina, San Diego, CA, USA). For the NGS data preprocessing, assembly, and reference mapping, the Geneious Prime (Version 2025.1) software was used. The nearly whole-genome sequences were submitted to the NCBI GenBank database. The name of the USUV strain (passage 2 isolate) is USUV-HUN-SM2024, and the GenBank Accession number is PQ772639. Based on GenBank records, detailed information on all USUV sequences obtained during the 2024 transmission season in Hungary is summarized in Supplementary File 3.

Phylogenetic analysis was based on nearly whole-genome sequences of the USUV strains. Pairwise alignment of the viral sequences was performed using Nucleotide BLAST (Basic Local Alignment Search Tool; https://blast.ncbi.nlm.nih.gov/Blast.cgi). For phylogenetic analysis, multiple sequence alignment was conducted using MAFFT (Multiple Alignment using Fast Fourier Transform; version 7: https://mafft.cbrc.jp/alignment/server/index.html). The phylogenetic tree was constructed with MEGA11 (Molecular Evolutionary Genetics Analysis, version 11). Maximum Likelihood analysis was performed under the General Time Reversible model with gamma-distributed rate variation among sites and a proportion of invariant sites (GTR + G + I). Bootstrap support values were estimated using 1,000 replicates.

### Usutu virus phylogeny and spread in Hungary, 2024

The co-circulation of different USUV lineages in Europe has been well documented in recent years. To date, at least eight distinct phylogenetic lineages have been described: three African and five European lineages (EU1–EU5) [26]. However, the terminology for lineages is not yet fully standardized; for instance some studies refer to EU5, while others define it as the Middle Eastern clade [27]. Among the European lineages, the EU2 is the most prevalent in Central and Eastern Europe, including Austria, Germany, Hungary, Italy, and Romania [28, 29]. A study by *Zecchin et al.* revealed that in Northeastern Italy, USUV strains clustered into two distinct groups, designated as EU2-A and EU2-B sublineages. According to phylogeographic analysis, Italy played a key role in the spatial dissemination of European USUV lineages. For instance, the Emilia-Romagna region has been identified as a major source of viral spread to Austria and Germany [29]. Two introduction events from Austria to Hungary have been identified: one in the 1990s and another between 2013 and 2016 [27, 29]. The EU2-A sublineage is considered to have high neurovirulence and has been dominant in recent outbreaks [30].

**Fig. 2:**
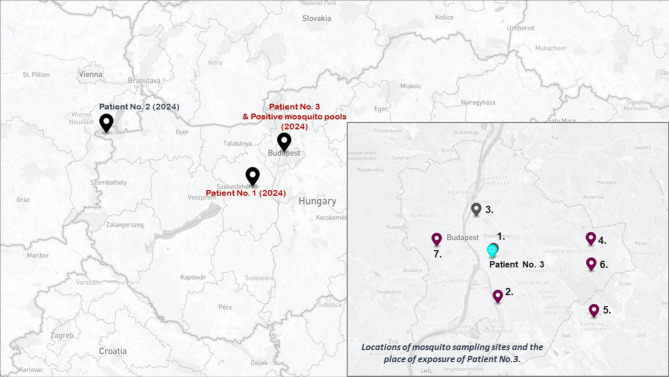
Geographical locations of confirmed human USUV infections and mosquito sampling sites in 2024. Whole genome USUV sequences are available from Patient No.1 and Patient No.3 (marked in red). Mosquito collection and USUV screening were carried out in Budapest. The locations of mosquito trapping sites, where USUV RT-qPCR pools were identified are marked with purple dots. The place of exposure of the immunocompromised patient (Patient No.3) is marked with a light blue dot. The map was created using the online map editor geojson.io (Retrieved June 15, 2025, from https://geojson.io/#map=12.91/46.90257/17.6427).

**Fig. 3:**
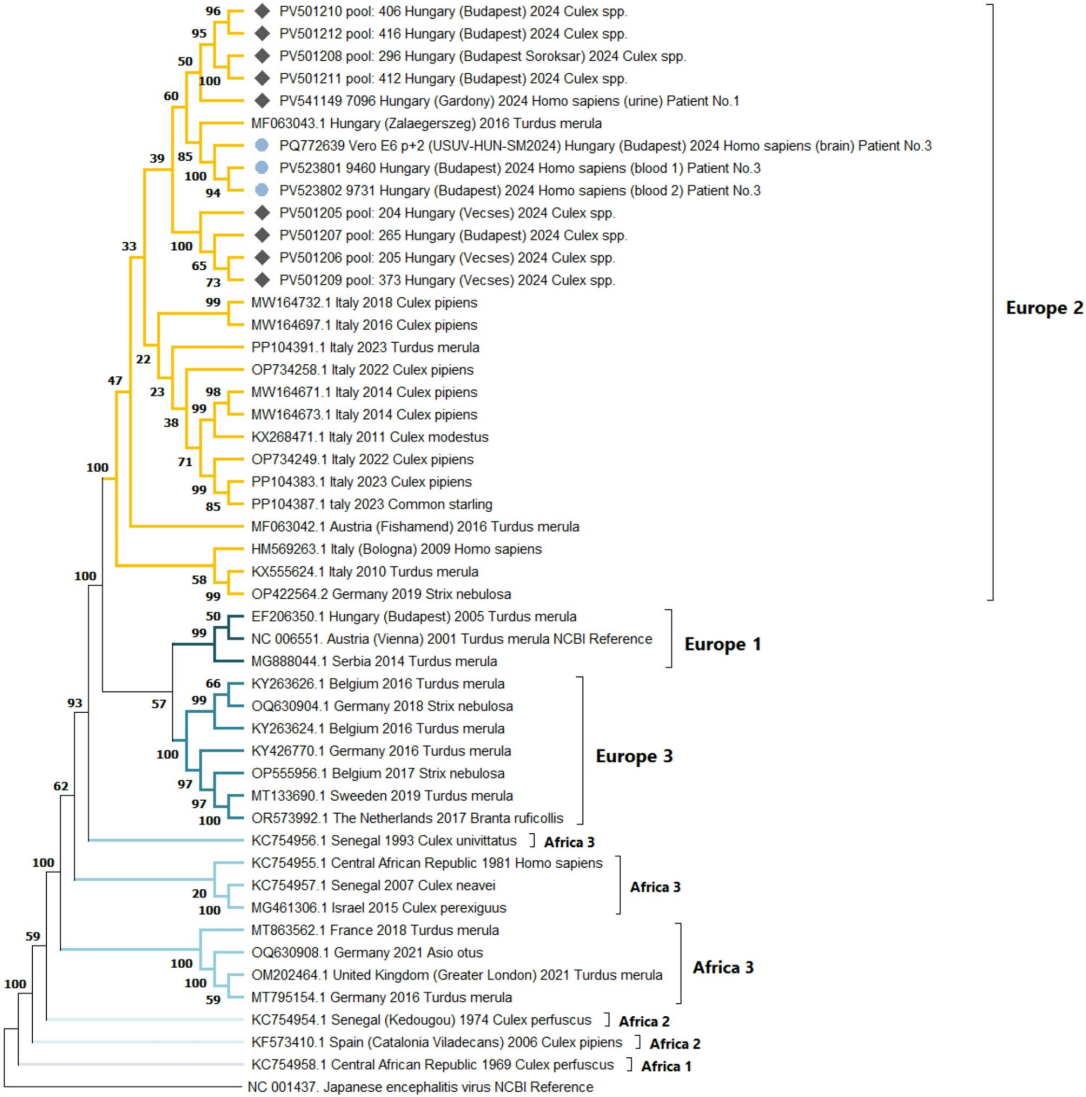
Maximum likelihood phylogenetic tree of nearly complete genome sequences of USUV strains. Sequence data are labeled with the following information: GenBank accession number, geographical location of virus isolation or detection, year of isolation or detection, and the scientific name of the host species. Sequences corresponding to the presented case are marked with light blue dots, while a dark blue diamond indicates sequences from another human case and eight mosquito pools collected in 2024

The virological differential diagnosis of WNV and USUV infections led to the identification of three laboratory-confirmed human USUV cases during the 2024 transmission period in Hungary. All three cases were associated with severe neurological manifestations. Symptom onset for the first case (Patient No. 1) occurred in early August, while the other two were diagnosed in September (Patient No. 2) and early October (Patient No. 3), respectively. The geographical distribution of the human USUV infections was relatively dispersed (Fig. 2). The presumed place of exposure for Patient No. 3 (presented in this study) is Budapest, as the patient was hospitalized at the National Institute of Hematology and Infectious Diseases during the incubation period. Between May 9 and November 8, 2024, as part of a pilot study, mosquito collection and pathogen surveillance were carried out at seven trapping sites in and around the capital city of Budapest (Fig. 2). A total of 7,410 female *Culex* spp. mosquitoes were collected and tested for USUV in 474 pools. USUV was detected in eight mosquito pools from five of the seven trapping sites. The first positive results were obtained from sampling sites No. 4 and No. 5 on July 21, while the last USUV-positive pool was identified at site No. 6 on August 25, 2024. All sampling sites and the locations of human exposure are illustrated in Fig. 2. USUV RNA was detected in clinical samples from Patients No. 1 and No. 3. Whole genome sequencing and subsequent phylogenetic analysis were conducted on all eight mosquito-positive pools, as well as the USUV-positive human specimens. Although mosquito surveillance was limited to a well-defined area, the combined evidence from mosquito and human infections demonstrates an active USUV circulation in Hungary. The resulting phylogenetic tree, shown in Fig. 3, revealed that Hungarian sequences clustered within European lineage 2.

In the 2024 Hungarian USUV sequences, two amino acid substitutions were identified: G595S in the E protein and E3425D in the NS5 protein; both mutations are characteristic of the EU2-A sublineage [28, 29]. Additional mutations specific to Italian strains [29] were also found in all Hungarian sequences: E830G (NS1 protein), I1324T (NS2A protein), and M2645I (NS5 protein). These findings are consistent with the phylogenetic analysis and confirm the close genetic relationship between Hungarian and Italian USUV strains (Fig. 3.). Notably, the G595S substitution is located within the DIII domain of the envelope protein and may play a role in the neuroinvasive potential of USUV in humans [31]. Most human neurological cases have been associated with the EU2 lineage, particularly the EU2-A sublineage, suggesting a potential link between the genetic characteristics and neurotropism [15]. However, this assumption has some limitations, such as the relatively small number of human cases and human-derived sequences, as well as the lack of detailed clinical data. Overall, the currently circulating EU2-A USUV strains in Hungary may pose a risk to human health, especially during periods of intense mosquito activity, which could lead to an increased number of human infections.

## Discussion

### Usutu virus– an emerging threat among immunocompromised patients?

This report presents the first documented autochthonous case of disseminated Usutu virus infection in a severely immunocompromised adult patient from Hungary, culminating in a fatal outcome. Diagnosing neuroinvasive arboviral infections in such individuals remains a clinical challenge due to the often misleading epidemiological history, along with nonspecific early symptoms, rapid disease progression, and diminished inflammatory responses. In addition, conventional diagnostic work-ups of viral encephalitis in European healthcare settings may not routinely include USUV, especially in patients without travel history or overt exposure to endemic vector habitats. The neuroinvasive character of the disease in our case was documented by progressive neurological deficits, deterioration into a comatose state, and findings from CSF analysis, EEG and cranial MRI. USUV RNA was detected in whole blood, urine, and CSF samples obtained on day + 4, as well as in *post-mortem* brain and bone marrow samples collected on day + 13, following symptom onset. Therapeutic attempts failed to attenuate disease progression. The case is comprehensively documented both from clinical and virological perspectives, and we feel that it may serve as a reference for future investigations into USUV pathogenesis among immunocompromised hosts.

**Fig. 4:**
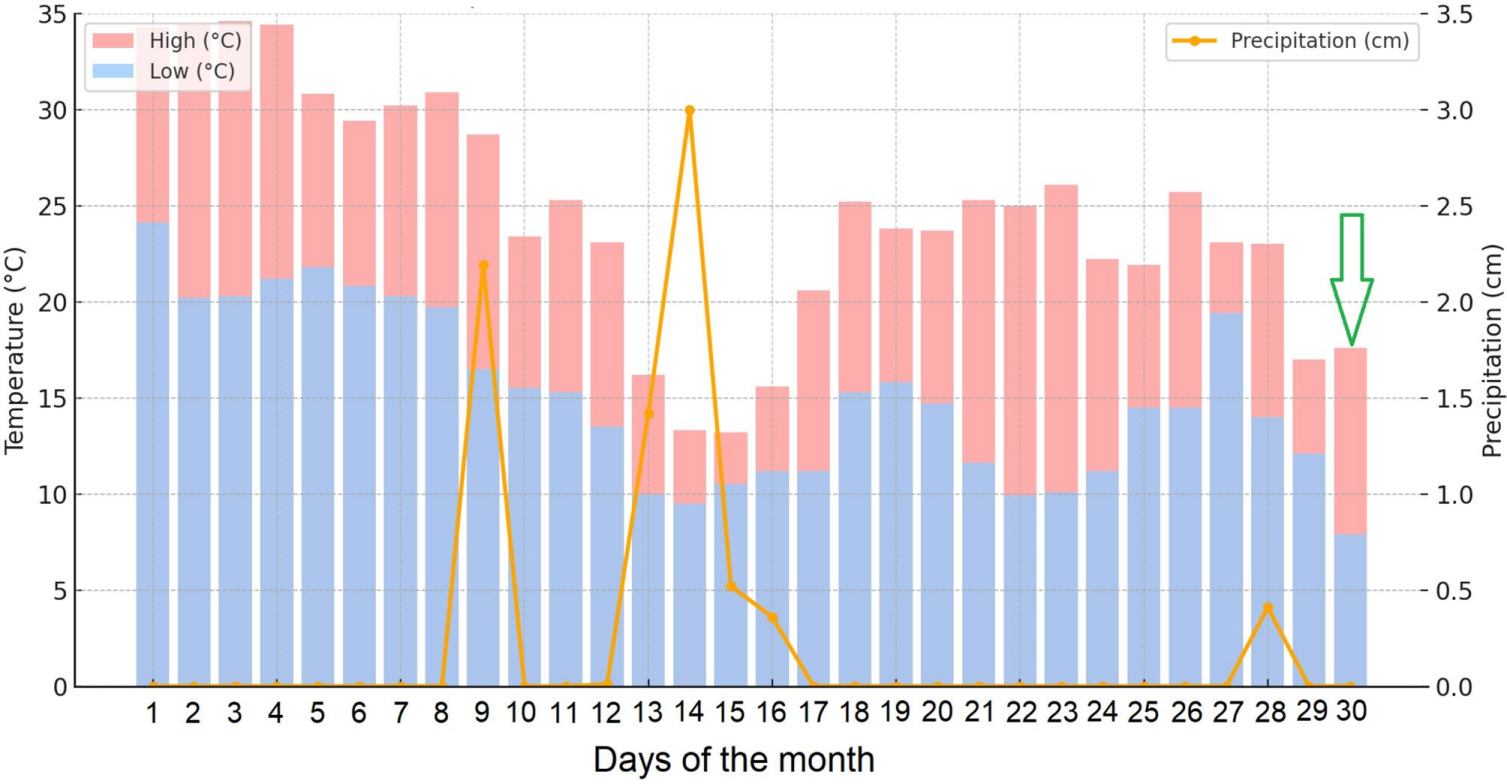
Daily actual high and low temperatures and precipitation in Budapest, Hungary. Parameters were recorded in the autumn season during the possible incubation period of the Usutu virus infection (3 to 12 days). The green arrow indicates the first day of symptom onset. The month is not specified for *General Data Protection Regulation* (GDPR) compliance. Data downloaded through Extreme Weather Watch (http://www.extremeweatherwatch.com/cities/budapest). Figure generated by ChatGPT 4.0

**Fig. 5:**
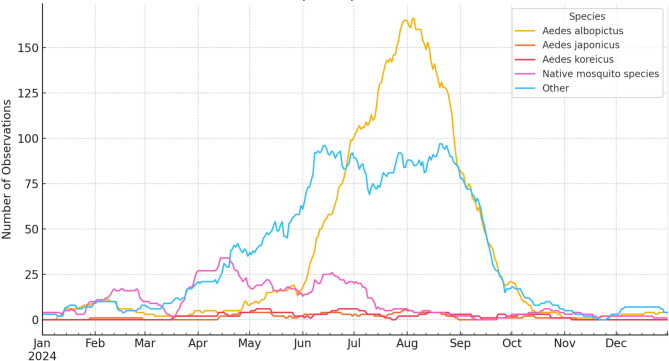
The projected monthly number of identified mosquito observations in 2024. Data reported through citizen science monitoring conducted by the Centre for Ecological Research (Budapest, Hungary), and downloaded through Mosquito Monitor (https://mosquitosurveillance.hu). Figure generated by ChatGPT 4.0

Given that the neutropenic patient was maintained in protective isolation throughout the presumed incubation period, the exact route of USUV infection remains uncertain. One plausible hypothesis is that, despite adherence to isolation protocols, an infected mosquito may have gained access to our patient’s environment. This assumption is supported by meteorological data indicating an average daily temperature of 24.1 °C (75.4 °F) and regional flooding due to increased precipitation (Fig. 4), and a concurrent rise in mosquito activity during the relevant timeframe (Figs. 4 and 5) [32]. The transmission dynamics of USUV are strongly influenced by environmental factors, particularly temperature and humidity. Warmer temperatures enhance mosquito reproduction, biting rates, and shorten the extrinsic incubation period of the virus, making transmission more efficient. In Africa, USUV is mostly detected during the rainy season, while in Europe, higher temperatures correlate with increased USUV transmission [33]. Although *Aedes albopictus*,* Ae. japonicus*, and *Ae. koreicus* are not the primary vectors of USUV, a significant increase in the activity of these invasive mosquitoes (Fig. 5), coinciding with favourable environmental conditions (Fig. 4), may have indicated a potential rise in the presence of other species, such as *Culex* spp. Alternatively, although less likely, transfusion-related transmission cannot be definitively excluded, as the patient received 54 units of red blood cells and 260 units of platelets within the 30 days preceding symptom onset.

To further assess the virulence of USUV among immunocompromised hosts, a literature search was conducted in PubMed, Scopus, and Embase databases from their inception until the end of 2024 using the search term “usutu*”. All retrieved records were screened for eligibility based on whether they reported individual cases of USUV infection in pediatric or adult immunocompromised patients. Articles were included if they provided patient-level data including, but not limited to, clinical characteristics, immune status, virological findings and outcomes [1, 11, 12, 34–36].

A total of seven individual cases of USUV infection among immunocompromised patients were identifiable from six publications between 2009 and 2023 (Table 3). Most cases were reported from Italy. The mean age of affected patients was approximately 60 years, with a balance between sexes. The underlying immunocompromising conditions included hematological malignancies in 4, solid tumors in 2, and solid organ transplantation in 1 case. Among hematological malignancies, chronic lymphocytic leukemia, diffuse large B-cell lymphoma, and allogeneic hematopoietic stem cell transplantation (allo-HSCT) were represented. One patient had undergone orthotopic liver transplantation and had a history of thrombotic thrombocytopenic purpura, while another had comorbid psoriasis and an infiltrating prostate adenocarcinoma. Finally, graft-versus-host disease was present in the post-HSCT case, also contributing to profound immunosuppression.

**Table 3:** Cases of immunocompromised patients with reported USUV infection in the literature with available patient-level data

First author	Publication year	Country	Patient number	Age, sex	Immuno-incompetence	Clinical characteristics	USUV lineage	Antiviral treatment	Patient outcome	PMID
*Cavrini et al.*	2009	Italy	1	40s^†^, female	Orthotopic liver transplantation and previous TTP	Encephalitis Fulminant hepatitis	EU1	n.d.	Survival	20,070,935
*Pecorari et al.*	2009	Italy	1	60s^†^, female	Diffuse large B-cell lymphoma receiving R-CHOP immuno-chemotherapy	Meningoencephalitis	n.a.	No	Survival	20,070,936
*Vilibic-Cavlek et al.*	2019	Croatia	1	60, n.d.	Chronic lymphocytic leukaemia	Neuroinvasive disease	EU2	n.d.	Death	31,067,011
*Pacenti et al.*	2019	Italy	2	60s^†^, male 90s^†^, male	Underlying malignancy Underlying malignancy	Aphasia, apraxia, disorientation Fever, headache, myalgia	EU2 EU2	No No	Survival Survival	31,771,697
*Zanella et al.*	2021	Switzerland	1	23, male	Allo-HSCT for lymphoma Gastrointestinal-skin-lung GvHD	Ongoing GvHD	n.d.	n.d.	Death	33,487,167
*Gaibani et al.*	2023	Italy	1	80, male	Infiltrating prostate adenocarcinoma and psoriasis	Septic shock Multi-organ failure	n.a.	No	Death	37,227,671
**TOTAL**	6 publications between 2009–2023	Italy: 5 Other: 2	7 patients	Approx. mean age 60 4 males	Hematological malignancy: 3 Other malignancy: 3 Solid organ transplantation: 1 Autoimmune disease:1	Neuroinvasive disease: 5 cases Hepatitis: 1 case Other: 2 cases	EU1: 1 case EU2: 3 cases n.d.: 3 cases	No: 4 cases n.d.: 3 cases	Survival: 4 cases Death: 3 cases	-

^†^ The authors reported the age decade

allo-HSCT: allogeneic hematopoietic stem cell transplantation, CNS: central nervous system, EU: Europe 2 lineage, GvHD: graft-versus-host disease, n.d.: no data, R-CHOP: rituximab, cyclophosphamid, doxorubicin, vincristin, prednisolone, TTP: thrombotic thrombocytopenic purpura, USUV: Usutu virus

Central nervous system involvement of neuroinvasion was the most common clinical manifestation, reported in five of the seven patients, and included presentations such as meningoencephalitis, aphasia, apraxia, and disorientation. One case presented with fulminant hepatitis, while two patients developed systemic complications such as septic shock or multi-organ failure. Documented USUV lineages included EU1 (*n* = 1) and EU2 (*n* = 3), while lineage data were not reported in three cases. None of the patients received specific antiviral treatment. Outcomes were variable: four patients survived and three patients died, corresponding to a calculated case fatality rate of approximately 43%. Due to the limited number of reported human USUV cases, determining accurate fatality rates among immunocompetent individuals remains difficult. Most human infections appear to be asymptomatic or present with only mild symptoms, indicating an underreporting of real case numbers. Furthermore, the majority of documented infections in Europe have been identified incidentally during routine WNV nucleic acid amplification testing of asymptomatic blood donors, due to cross-reactivity with USUV [6, 8–10, 37]. Notably, all fatalities occurred in patients with hematological malignancies or allo-HSCT and were associated with either neuroinvasive disease or multi-organ failure. These findings highlight the potential severity and an increased risk of fatal outcomes of USUV infection among immunocompromised patients.

### Potential pathogenesis and immune evasion of Usutu virus

In light of the documented poor clinical outcomes associated with USUV infection in immunocompromised patients, we investigated the published literature for potential mechanisms of pathogenesis and immune evasion that may contribute to this effect. As WNV and USUV have a close genetic relationship, USUV may utilize similar steps in vivo as WNV. The initial site of WNV infection is the skin, where the virus is thought to replicate in keratinocytes and Langerhans cells (LC). These specialized dendritic cells then migrate to the draining lymph nodes, initiating a primary viremia that leads to the subsequent infection of peripheral organs [38]. A recent study comparing USUV and WNV revealed that USUV demonstrated a greater ability to infect skin-resident dendritic cells, particularly epidermal LCs, as the proportion of infected LCs was approximately twice as high with USUV compared to WNV, and USUV showed a preference for epidermal over dermal dendritic cells. In addition, USUV replicated efficiently within LCs, with viral RNA levels around ten times higher than those observed in WNV-infected cells [39]. A study investigating the role of human skin as a key site for USUV replication and early immune response found that despite a robust early innate immune response, including the induction of type I and III IFNs, and interferon-stimulated genes such as RSAD2 and OAS1, USUV was still able to spread within the skin. This indicates that the virus can partially evade host immune defenses, particularly in the first 24 h post-infection [40]. These findings might indicate that USUV has a distinct tropism for LCs and may establish infection more effectively at the skin entry site.

Detailed knowledge of USUV neuroinvasion mechanisms remains limited. To better understand the role of USUV in neurological impairments, the neurotropism of a European USUV strain (Vienna-2001) using both in vivo and ex vivo murine models was examined. Acute hippocampal slices from wild-type mouse brains were infected with USUV, and analysis at four days post-infection revealed viral replication and broad neurotropism to multiple CNS cell types, including neurons, astrocytes, and microglia [41]. In addition, in vitro studies using a human blood-brain barrier (BBB) model showed that USUV infection leads to upregulation and release of inflammatory cytokines such as IL-6, TNF-α, and IL-1β, both on the apical and basolateral sides of the barrier. Despite inducing inflammation, USUV causes only modest disruption of the BBB integrity. USUV infection also stimulated the secretion of chemoattractants such as CCL2, CXCL10, and CCL5, promoting immune cell recruitment to the CNS. Furthermore, USUV-infected endothelial cells exhibited upregulation of cell adhesion molecules (e.g., ICAM-1, VCAM-1, PECAM, selectins), facilitating the subsequent interaction with these recruited immune cells. Interestingly, USUV showed a higher replication rate in endothelial cells compared to WNV, but induced a less intense inflammatory response, suggesting a lower capacity for immune-mediated damage. Overall, while USUV can replicate efficiently in BBB cells and promote immune activation, its impact on neuroinflammation appears milder than that of WNV [42].

### Opportunities for treatment and prevention of human Usutu virus infections

Although as of 2025, there is no specific antiviral treatment for USUV, some preclinical data are suggesting potential efficacy of broad-spectrum antivirals and immunotherapeutic approaches. Favipiravir, a viral RNA polymerase inhibitor, has demonstrated efficacy in animal models. *Segura Guerrero et al.*. confirmed that favipiravir inhibits USUV replication in vitro in Vero E6 cells, particularly when administered early during the infection process [43]. To further assess the effectiveness of the drug, a murine model using AG129 mice lacking type I and II interferon receptors was developed. While USUV infection within this model led to fulminant infection and death within a few days, treatment with favipiravir in infected mice delayed disease progression, reduced viral loads in the blood and extended survival time. However, it did not fully prevent mortality, especially at higher viral loads [16, 43]. In addition, a study revealed a critical interaction between the USUV NS5 protein and the host cell kinase Akt, as NS5 is shown to be phosphorylated by Akt, suggesting that this post-translational modification may regulate its activity. Using specific Akt inhibitors, like ipatasertib and MK-2206, the study demonstrated a significant reduction in USUV replication in cultured cells. Additionally, honokiol, a broader inhibitor of the PI3K/Akt/mTOR axis, exhibited even stronger antiviral activity, although its lack of specificity raises concerns about off-target effects. These findings might support the hypothesis of targeting Akt or its associated signalling pathways as a potential therapeutic strategy against USUV [44]. Finally, a recent study examined the antiviral efficacy of interferon-beta (IFN-β) and 2′C-methylcytidine (2CMC), a synthetic nucleoside analogue that targets flaviviral RNA-dependent RNA polymerase, using human fetal organotypic brain slice cultures. Both agents demonstrated significant inhibitory effects on USUV replication in this model [45].

As the primary mode of USUV transmission is through mosquito bites, preventive strategies focus on mosquito control and personal protection. This includes the use of insect repellents, mosquito nets, and insecticides, as well as eliminating stagnant water around homes and healthcare facilities to prevent mosquito breeding potential [16]. Following the detection of USUV in asymptomatic blood donors, Italian health authorities implemented nucleic acid testing for all blood donations in the affected area. This intervention was crucial in preventing potential transfusion-transmitted infections, particularly among immunocompromised recipients [46]. Despite that currently there are no approved vaccines for USUV, studies by *Wang et al.* and *Jurišić et al.* might highlight promising advances in vaccine development against USUV. *Wang et al.* developed a live-attenuated chimeric vaccine by replacing the prM and E structural genes of a dengue virus type 2 vaccine strain with those from an European USUV strain, generating replication in Vero cells, strong neutralizing antibody responses, and complete protection against lethal USUV challenge in both immunocompetent and immunodeficient mice [47]. Complementing this, *Jurišić et al.* investigated both live-attenuated USUV strains and a chimeric WNV expressing the USUV envelope protein, inducing immune responses protective against lethal USUV infection in mice [48]. Both experimental studies may highlight a future potential for controlling multiple co-circulating arboviruses [47, 48].

### Limitations and research gaps

Our report only documents a single case, restricting generalization. While comprehensive clinical, virological, and radiological data were collected, the absence of a control group or a standardized treatment protocol limits the ability to draw direct causality regarding disease progression and therapeutic response. There are significant gaps in current knowledge about USUV infection, such as the exact steps of human pathogenesis on a subcellular and cellular level, the clinical spectrum of tissue invasivity, and optimal therapeutic strategies among immunosuppressed populations. While the virus has been increasingly detected in avian and mosquito samples across Europe, human cases remain rarely reported, and prospective data are lacking. Further research is required to assess host-pathogen interactions in immunocompromised individuals, identify clinical predictors of poor outcome, and explore the utility of possible antiviral treatments. Multi-national surveillance and the establishment of a case registry would be crucial in improving our understanding of this emerging neurotropic arbovirus.

## Conclusion

Usutu virus infection, an emerging neuroinvasive arboviral disease, could be associated with clinical complications and poor outcomes among immunocompromised hosts. Diagnostic algorithms for Usutu virus in endemic regions, particularly during peak mosquito seasons in Central and Eastern Europe, and a call for awareness and research might ultimately reduce morbidity and mortality in vulnerable patient populations.

## Supplementary Information

Below is the link to the electronic supplementary material.

Supplementary Material 1

Supplementary Material 2

Supplementary Material 3

## Data Availability

The nearly whole-genome sequences were submitted to the NCBI GenBank database. The name of the USUV strain (passage 2 isolate) is USUV-HUN-SM2024, and the GenBank Accession number is PQ772639.

## Abbreviations

AMMoL: Acute myelomonocytic leukemia
ATCC: American Type Culture Collection
BBB: Blood-brain barrier
CMV: Cytomegalovirus
CNS: Central nervous system
CSF: Cerebrospinal fluid
Ct: Cycle threshold
CT: Computed tomography
CV: Coefficient of variation
DNA: Deoxyribonucleic acid
EEG: Electroencephalography
FLAG-M: Fludarabine, cytarabine, mitoxantrone
HSCT: Hematopoietic stem cell transplantation
ICU: Intensive care unit
IFA: Indirect immunofluorescence assay
IgA: Immunoglobulin A
IgG: Immunoglobulin G
IgM: Immunoglobulin M
IVIG: Intravenous immunoglobulin
JCV: JC virus
JEV: Japanese encephalitis virus
LC: Langerhans cells
LoD: Limit of detection
MRI: Magnetic resonance imaging
NGS: Next-generation sequencing
PCR: Polymerase-chain reaction
RNA: Ribonucleic acid
RT-qPCR: Reverse transcription real-time PCR
TBEV: Tick-borne encephalitis virus
USUV: Usutu virus
WNV: West Nile virus

## References

[1] Pacenti M, Sinigaglia A, Martello T, de Rui ME, Franchin E, Pagni S, et al. Clinical and virological findings in patients with Usutu virus infection, Northern italy, 2018. Euro Surveill. 2019;24(47):1900180. 31771697 10.2807/1560-7917.ES.2019.24.47.1900180 PMC6885746

[2] Nikolay B, Diallo M, Boye CSB, Sall AA. Usutu virus in Africa. Vector Borne Zoonotic Dis. 2011;11(11):1417–23. 21767160 10.1089/vbz.2011.0631

[3] Vilibic-Cavlek T, Petrovic T, Savic V, Barbic L, Tabain I, Stevanovic V, et al. Epidemiology of Usutu virus: the European scenario. Pathogens. 2020;9(9):1–19. 10.3390/pathogens9090699 PMC7560012 32858963

[4] Clé M, Beck C, Salinas S, Lecollinet S, Gutierrez S, Van de Perre P, et al. Usutu virus: a new threat? Epidemiol Infect. 2019;147:1–11. 10.1017/S0950268819001213 PMC6625183 31364580

[5] Gaibani P, Rossini G. An overview of Usutu virus. Microbes Infect. 2017;19(7–8):382–7. 28602915 10.1016/j.micinf.2017.05.003

[6] Bakonyi T, Jungbauer C, Aberle SW, Kolodziejek J, Dimmel K, Stiasny K, et al. Usutu virus infections among blood donors, austria, July and August 2017– raising awareness for diagnostic challenges. Euro Surveill. 2017;22(41):17–8. 10.2807/1560-7917.ES.2017.22.41.17-00644 PMC5710119 29043962

[7] Čabanová V, Kerlik J, Kirschner P, Rosochová J, Klempa B, Sláviková M, et al. Co-circulation of West Nile, Usutu, and tick-borne encephalitis viruses in the same area: a great challenge for diagnostic and blood and organ safety. Viruses. 2023;15(2):1–27. 10.3390/v15020366 PMC9966648 36851580

[8] Cadar D, Maier P, Müller S, Kress J, Chudy M, Bialonski A, et al. Blood donor screening for West Nile virus (WNV) revealed acute Usutu virus (USUV) infection, Germany, September 2016. Euro Surveill. 2017;22(14):pii–30501. 10.2807/1560-7917.ES.2017.22.14.30501 PMC5388121 28422005

[9] Zaaijer HL, Slot E, Molier M, Reusken CBEM, Koppelman MHGM. Usutu virus infection in Dutch blood donors. Transfusion. 2019;59(9):2931–7. 31270821 10.1111/trf.15444

[10] Angeloni G, Bertola M, Lazzaro E, Morini M, Masi G, Sinigaglia A, et al. Epidemiology, surveillance and diagnosis of Usutu virus infection in the EU/EEA, 2012 to 2021. Euro Surveill. 2023;28(33):2200929. 37589592 10.2807/1560-7917.ES.2023.28.33.2200929 PMC10436690

[11] Pecorari M, Gennari W, Grottola A, Sabbatini A, Tagliazucchi S, Savini G, et al. First human case of Usutu virus neuroinvasive infection, Italy, August–September 2009. Euro Surveill. 2009;14(50):pii–19446. 20070936

[12] Cavrini F, Gaibani P, Longo FG, Pierro AM, Rossini GP, Bonilauri P, et al. Usutu virus infection in a patient who underwent orthotopic liver transplantation, italy, August–September 2009. Euro Surveill. 2009;14(50):pii–19448. 20070935

[13] Simonin Y. Circulation of West Nile virus and Usutu virus in Europe: overview and challenges. Viruses. 2024;16(4):1–15. 10.3390/v16040599 PMC11055060 38675940

[14] Clé M, Barthelemy J, Desmetz C, Foulongne V, Lapeyre L, Bolloré K, et al. Study of Usutu virus neuropathogenicity in mice and human cellular models. PLoS Negl Trop Dis. 2020;14(4):e0008153. 32324736 10.1371/journal.pntd.0008223 PMC7179837

[15] Clé M, Constant O, Barthelemy J, Desmetz C, Martin MF, Lapeyre L, et al. Differential neurovirulence of Usutu virus lineages in mice and neuronal cells. J Neuroinflammation. 2021;18(1):1–14. 33407600 10.1186/s12974-020-02060-4 PMC7789689

[16] Cadar D, Simonin Y. Human Usutu virus infections in Europe: a new risk on horizon? Viruses. 2022;15(1):77. 10.3390/v15010077 PMC9866956 36680117

[17] Nagy A, Mezei E, Nagy O, Bakonyi T, Csonka N, Kaposi M, et al. Extraordinary increase in West Nile virus cases and first confirmed human Usutu virus infection in Hungary, 2018. Euro Surveill. 2019;24(28):1900038. 31311619 10.2807/1560-7917.ES.2019.24.28.1900038 PMC6636212

[18] Nagy A, Csonka N, Takács M, Mezei E, Barabás É. West Nile and Usutu virus seroprevalence in Hungary: a nationwide serosurvey among blood donors in 2019. PLoS ONE. 2022;17(4):e0266632. 35395048 10.1371/journal.pone.0266840 PMC8992992

[19] Bakonyi T, Erdélyi K, Ursu K, Ferenczi E, Csörgo T, Lussy H, et al. Emergence of Usutu virus in Hungary. J Clin Microbiol. 2007;45(12):3870–4. 17913929 10.1128/JCM.01390-07 PMC2168571

[20] European Centre for Disease Prevention and Control. West Nile virus infections in 2024. 2024 [cited 2025 Jun 15]. Available from: https://www.ecdc.europa.eu/en/west-nile-fever/surveillance-and-disease-data/historical

[21] Hungarian Ministry of Health. Regulation No. 18/1998 (VI. 3.) of the Ministry of Health on epidemiological measures necessary for the prevention of infectious diseases and epidemics. 1998 [cited 2025 Jun 11]. Available from: https://net.jogtar.hu/jogszabaly?docid=99800018.nm

[22] Schwaiger M, Cassinotti P. Development of a quantitative real-time RTPCR assay with internal control for the laboratory detection of tick borne encephalitis virus (TBEV) RNA. J Clin Virol. 2003;27(2):136–45. 12829035 10.1016/s1386-6532(02)00168-3

[23] Nikolay B, Weidmann M, Dupressoir A, Faye O, Boye CS, Diallo M, et al. Development of a Usutu virus specific real-time reverse transcription PCR assay based on sequenced strains from Africa and Europe. J Virol Methods. 2014;197:51–4. 24036076 10.1016/j.jviromet.2013.08.039

[24] Nagy A, Szöllősi T, Takács M, Magyar N, Barabás É. West Nile virus seroprevalence among blood donors in Hungary. Vector Borne Zoonotic Dis. 2019;19(11):844–50. 31184991 10.1089/vbz.2018.2401

[25] Chrzastek K, Lee DH, Smith D, Sharma P, Suarez DL, Pantin-Jackwood M, et al. Use of Sequence-Independent, Single-Primer-Amplification (SISPA) for rapid detection, identification, and characterization of avian RNA viruses. Virology. 2017;509:159–66. 28646651 10.1016/j.virol.2017.06.019 PMC7111618

[26] Cadar D, Lühken R, Van Der Jeugd H, Garigliany M, Ziegler U, Keller M, et al. Widespread activity of multiple lineages of Usutu virus, Western Europe, 2016. Euro Surveill. 2017;22(4):30452. 28181903 10.2807/1560-7917.ES.2017.22.4.30452 PMC5388094

[27] Siljic M, Sehovic R, Jankovic M, Stamenkovic G, Loncar A, Todorovic M, et al. Evolutionary dynamics of Usutu virus: worldwide dispersal patterns and transmission dynamics in Europe. Front Microbiol. 2023;14:1145981. 37032910 10.3389/fmicb.2023.1145981 PMC10076808

[28] Prioteasa FL, Dinu S, Tiron GV, Stancu IG, Fălcuță E, Ceianu CS, et al. First detection and molecular characterization of Usutu virus in culex pipiens mosquitoes collected in Romania. Microorganisms. 2023;11(3):684. 36985256 10.3390/microorganisms11030684 PMC10054730

[29] Zecchin B, Fusaro A, Milani A, Schivo A, Ravagnan S, Ormelli S, et al. The central role of Italy in the spatial spread of Usutu virus in Europe. Virus Evol. 2021;7(1):veab020. 34513027 10.1093/ve/veab048 PMC8427344

[30] Weidinger P, Kolodziejek J, Bakonyi T, Brunthaler R, Erdélyi K, Weissenböck H, et al. Different dynamics of Usutu virus infections in Austria and Hungary, 2017–2018. Transbound Emerg Dis. 2020;67(1):298–307. 31505099 10.1111/tbed.13351 PMC7003936

[31] Gaibani P, Cavrini F, Gould EA, Rossini G, Pierro A, Landini MP, et al. Comparative genomic and phylogenetic analysis of the first Usutu virus isolate from a human patient presenting with neurological symptoms. PLoS ONE. 2013;8(5):e64761. 23741387 10.1371/journal.pone.0064761 PMC3669420

[32] Garamszegi LZ, Kurucz K, Soltész Z. Validating a surveillance program of invasive mosquitoes based on citizen science in Hungary. J Appl Ecol. 2023;60(7):1481–94.

[33] Morozińska-Gogol J. Mosquito borne virus USUTU as potential threat to human health. Ann Parasitol. 2024;70(2):1–17. 39097293 10.17420/ap7002.524

[34] Gaibani P, Barp N, Massari M, Negri EA, Rossini G, Vocale C, et al. Case report of Usutu virus infection in an immunocompromised patient in italy, 2022. J Neurovirol. 2023;29(3):364–6. 37227671 10.1007/s13365-023-01148-w PMC10211289

[35] Vilibic-Cavlek T, Savic V, Petrovic T, Toplak I, Barbic L, Petric D, et al. Emerging trends in the epidemiology of West Nile and Usutu virus infections in Southern Europe. Front Vet Sci. 2019;6:437. 31867347 10.3389/fvets.2019.00437 PMC6908483

[36] Zanella MC, Cordey S, Laubscher F, Docquier M, Vieille G, Van Delden C, et al. Unmasking viral sequences by metagenomic next-generation sequencing in adult human blood samples during steroid-refractory/dependent graft-versus-host disease. Microbiome. 2021;9(1):28. 33487167 10.1186/s40168-020-00953-3 PMC7831233

[37] Frank C, Schmidt-Chanasit J, Ziegler U, Lachmann R, Preußel K, Offergeld R. West Nile virus in germany: an emerging infection and its relevance for transfusion safety. Transfus Med Hemother. 2022;49(4):192–204. 36159956 10.1159/000525167 PMC9421668

[38] Agliani G, Giglia G, Marshall EM, Gröne A, Rockx BHG, van den Brand JMA. Pathological features of West Nile and Usutu virus natural infections in wild and domestic animals and in humans: a comparative review. One Health. 2023;16:100510. 37363223 10.1016/j.onehlt.2023.100525 PMC10288044

[39] Martin MF, Maarifi G, Abiven H, Seffals M, Mouchet N, Beck C, et al. Usutu virus escapes langerin-induced restriction to productively infect human Langerhans cells, unlike West Nile virus. Emerg Microbes Infect. 2022;11(1):761–74. 35191820 10.1080/22221751.2022.2045875 PMC8903762

[40] Vouillon A, Barthelemy J, Lebeau L, Nisole S, Savini G, Lévêque N, et al. Skin tropism during Usutu virus and West Nile virus infection: an amplifying and immunological role. J Virol. 2024;98(1):e01536–23. 10.1128/jvi.01830-23 PMC10805065 38088560

[41] Salinas S, Constant O, Desmetz C, Barthelemy J, Lemaitre JM, Milhavet O, et al. Deleterious effect of Usutu virus on human neural cells. PLoS Negl Trop Dis. 2017;11(9):e0005913. 28873445 10.1371/journal.pntd.0005913 PMC5600396

[42] Constant O, Maarifi G, Barthelemy J, Martin MF, Tinto B, Savini G, et al. Differential effects of Usutu and West Nile viruses on neuroinflammation, immune cell recruitment and blood–brain barrier integrity. Emerg Microbes Infect. 2023;12(1):1–12. 10.1080/22221751.2022.2156815 PMC9815434 36495563

[43] Segura Guerrero NA, Sharma S, Neyts J, Kaptein SJF. Favipiravir inhibits in vitro Usutu virus replication and delays disease progression in an infection model in mice. Antiviral Res. 2018;160:137–42. 30385306 10.1016/j.antiviral.2018.10.026

[44] Albentosa-González L, Sabariegos R, Arias A, Clemente-Casares P, Mas A. Akt interacts with Usutu virus polymerase, and its activity modulates viral replication. Pathogens. 2021;10(2):1–15. 10.3390/pathogens10020244 PMC7924047 33672588

[45] Marshall EM, Rashidi AS, van Gent M, Rockx B, Verjans GMGM. Neurovirulence of Usutu virus in human fetal organotypic brain slice cultures partially resembles Zika and West Nile virus. Sci Rep. 2024;14(1):11223. 39209987 10.1038/s41598-024-71050-w PMC11362282

[46] Caracciolo I, Mora-Cardenas E, Aloise C, Carletti T, Segat L, Burali MS, et al. Comprehensive response to Usutu virus following first isolation in blood donors in the Friuli Venezia Giulia region of italy: development of Recombinant NS1-based serology and sensitivity to antiviral drugs. PLoS Negl Trop Dis. 2020;14(3):e0008088. 32226028 10.1371/journal.pntd.0008156 PMC7145266

[47] Wang ZJ, Zhang RR, Wu M, Zhao H, Li XF, Ye Q, et al. Development of a live-attenuated chimeric vaccine against the emerging Usutu virus. Vaccine. 2024;42(6):1363–71. 38310016 10.1016/j.vaccine.2024.01.077

[48] Jurisic L, Malatesta D, Zaccaria G, Di Teodoro G, Bonfini B, Valleriani F, et al. Immunization with Usutu virus and with a chimeric West Nile virus (WNV) harboring Usutu-E protein protects immunocompetent adult mice against lethal challenges with different WNV lineage 1 and 2 strains. Vet Microbiol. 2023;277:109604. 10.1016/j.vetmic.2022.109636 36580873

